# Vincent’s Angina

**Published:** 1918-08

**Authors:** 


**Vincent's Angina Infection : Its Prevalence, Varied Manifestations, Treatment, and Bacteriology.** By Horace Greeley, *American Journal of Medical Sciences.*

In this article, Greeley mentions that Taylor and McKinstry[1] reported that during the last few months over 300 cases of the malady had been confirmed bacteriologically. Quoting Bonby[2] he stated that in time of peace the disease constitutes about 2 or 3 o/o of all cases of throat complaints, among the French Army. Recent statistics from a British Military Hospital in France show the proportion to be as high as 23 o/o of all throat complaints. He believes that there are sufficient reports to indicate that the malady is of universal distribution although severe cases are not very common under normal conditions in civil practice. Among civilians he has noticed an apparent increase in the number of tonsillar and pharyngeal smears both in hospital and private practice that show the fusiform sperelleform organism.

*Predisposing Causes* : General debilitating diseases — viz., extreme fatigue, chilling, improper food — and excessive alcohol. Local influences are decayed teeth, excessive smoking, or chewing of tobacco.

*Exciting Cause* : Undoubtedly the organism so constantly associated with the lesions.

*Location and Appearence of Lesions* : Heavy dirty looking membrane covering one of the tonsils or any place upon the nasopharyngeal or buccal mucous membrane. It may extend over most of the surface of the upper respiratory tract.

*Symptoms* : Headache; malaise; glandular swelling adjoining the ulcerations; slight temperature; membrane which forms usually after symptoms have lasted for several days.

Severe forms reported from the European Military Hospitals have manifested great prostration, high fever, and albuminuria.

*Treatment* : Preventive — teeth put in best possible condition — avoidance of tobacco.

1. *Brit. Med. Jour.*, March 31, 1917.
2. *Brit. Med. Jour.*, Nov. 24, 1917.

